# Dynamic Channel Characteristic Analysis and Modeling of Conductive Intracardiac Communication Based on Sinusoidal Response and Impulse Response

**DOI:** 10.3390/bioengineering13060628

**Published:** 2026-05-27

**Authors:** Yu Chen, Yong Xu, Ya Zhou, Xuce Fan, Chang Yang, Yunjia Ge, Yong Song

**Affiliations:** 1 School of Optics and Photonics, Beijing Institute of Technology, Beijing 100081, China; chenyu_bit@163.com (Y.C.); zhouya@bit.edu.cn (Y.Z.); fanxc1203@163.com (X.F.); 3120230603@bit.edu.cn (C.Y.); 13683344276@163.com (Y.G.); 2 Senior Department of Cardiology, the Sixth Medical Center of PLA General Hospital, Beijing 100048, China; yongxu301@163.com

**Keywords:** conductive intracardiac communication, dynamic channel characteristics, channel parameters, impulse response

## Abstract

Conductive intracardiac communication (CIC) is one of the most innovative and promising communication technologies in multi-point cardiac pacing schemes that utilize the heart as the transmission channel in recent years. Current research predominantly focuses on static channel characteristics. Although some studies have explored dynamic responses, they are largely confined to basic amplitude–frequency and amplitude–time behaviors, lacking in-depth analysis of underlying dynamic mechanisms such as path loss, shadowing, multipath, and Doppler effects. Designing CIC systems solely on the basis of static properties can result in inaccurate channel estimation, distorted channel state information (CSI), and elevated bit error rate (BER). To solve the problems of dynamic channel measurement and modeling, this paper for the first time proposes a dynamic channel modeling method for CIC based on sinusoidal response and impulse response. Firstly, we develop a physical simulation and miniaturized measurement setup to measure the dynamic cardiac channel, and analyze the amplitude–frequency characteristics and amplitude–time characteristics. The influence of factors such as instrument differences, heart rate, flow rate, and motion artifacts is also discussed. Secondly, we systematically analyze the path loss, shadowing effect, multipath effect, and Doppler effect of the CIC channel. Combined with the dynamic channel characteristics and parameters, we propose a composite fading dynamic channel model and analyze the BER performance of baseband signal transmission and On–Off Keying (OOK) modulation systems. We conclude that (1) the CIC channel exhibits capacitive characteristics. Fixed electrodes can effectively suppress motion artifacts. (2) The dynamic channel gain of CIC varies periodically with the heartbeat, and the fluctuation range of the signal is less than 1–2 dB. (3) The dynamic CIC channel presents extremely weak shadow fading, no significant multipath, and no measurable Doppler characteristics, belonging to an extremely slow-fading channel. This work provides effective dynamic channel measurement approaches and a parameter basis for the transceiver design of CIC and a reliable model for the simulation of CIC systems.

## 1. Introduction

Cardiovascular disease remains the leading cause of death globally, claiming an estimated 19.2 million lives annually and accounting for approximately one-third of all deaths worldwide. Leadless cardiac pacemakers (LCPs) represent a minimally invasive therapy for cardiac rhythm disorders, delivering electrical pulses to stimulate myocardial contraction without transvenous leads. In dual-chamber systems, two pacemakers implanted in the right atrium and right ventricle coordinate contractions through conductive intracardiac communication (CIC) to maintain normal cardiac pumping function [1].

**Figure 1:**
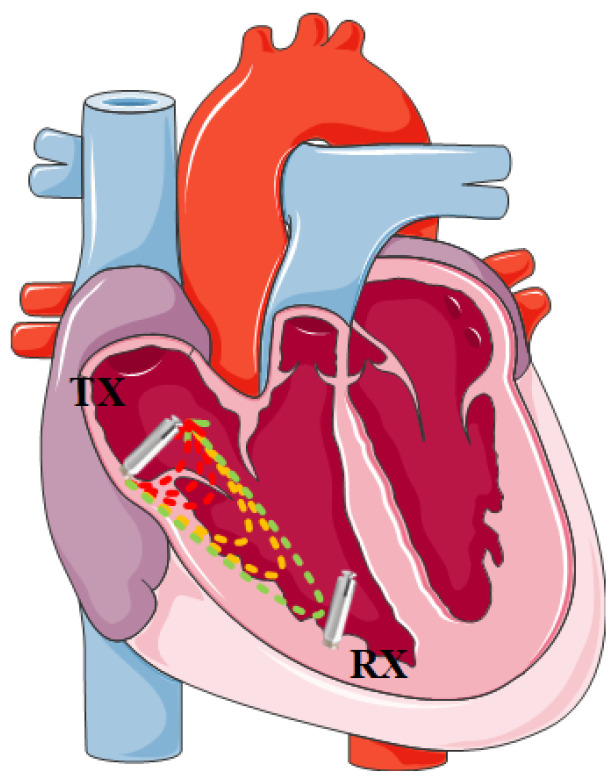
Principle schematic of conductive intracardiac communication. One pacemaker (TX) is placed in the upper or middle part of the right atrium, and the other (RX) is placed at the apex of the right ventricle.

CIC is a short-range implantable intra-body communication technology that exploits the electrical conductivity of myocardial tissue and blood to transmit signals between pacemaker electrodes. As illustrated in Figure 1, the majority of the injected current returns to the negative electrode at the transmitting end, while a small fraction is captured by the positive and negative electrodes at the receiving end [2]. Compared with conventional radio-frequency communication, CIC offers superior data privacy and substantially lower power consumption, making it particularly attractive for implantable cardiac applications [3].

The majority of existing CIC research relies on static modeling and simulation approaches. Maldari et al. employ finite-element models (FEM) to clarify channel attenuation from 40 kHz to 20 MHz [4]. Noormohammadi et al. incorporate wideband tissue properties at 300 MHz–500 MHz using the HUGO model in CST Microwave Studio [5], and Liu et al. develop a variable-volume heart model [6].

However, these static approaches inherently assume time-invariant channel conditions. When analyzing heartbeat-induced variations, the cardiac cycle is artificially segmented into discrete phases, and data are collected only after deformation stabilizes [7]. Consequently, such models provide outdated channel state information. Additionally, modifying a complex and detailed cardiac electromagnetic model is quite difficult, and the simulation requires extremely high computing power, with the simulation time being too long [8,9].

More recent efforts begin to explore time-varying channel behaviors. Bereuter et al. experimentally determine amplitude–frequency characteristics below 1 MHz [10,11,12]. Khaleghi et al. design a pulse signal generator and capture dynamic channel gains from 1 MHz to 15 MHz in in vivo animal experiments [13]. Chen et al. develop an ex vivo heart-beating simulator using a peristaltic pump to perfuse isolated hearts, measuring single-cycle attenuation curves and channel gain variations across 10 kHz–10 MHz while clarifying the effects of heart volume, blood content, and flow rate [14].

Regarding circuit modeling, Ziliang Wei et al. establish a time-varying circuit model based on cardiac mechanics [15], while Dongming Li et al. propose a model simulating both healthy and diseased hearts [16] and introduce a dynamic gain compensation method [17]. Han Wang et al. design a high common-mode rejection measurement circuit and construct an equivalent circuit model for cardiac biomechanical impedance [18].

Despite these advances, existing studies primarily focus on amplitude–frequency and amplitude–time characteristics. Fundamental dynamic fading phenomena including shadow fading, multipath propagation, and Doppler frequency shifts have not been systematically investigated or experimentally validated under dynamic cardiac conditions. A comprehensive dynamic channel model capable of guiding communication system design remains absent.

To address these gaps, the main contributions of this paper are summarized as follows:

(1) A novel dynamic channel physical simulation and miniaturized measurement device is developed, enabling the systematic separation of motion artifacts from real channel fading in ex vivo experiments.

(2) Experimental results verify that the dynamic CIC channel exhibits extremely weak shadow fading, negligible multipath, and no measurable Doppler shift, classifying it as an extremely slow-fading channel.

(3) A composite fading model for CIC channels is proposed along with its key parameters, establishing a theoretical framework for dynamic characterization.

The remainder of this paper is organized as follows. Section 2 describes the measurement methods and principles. Section 3 analyzes the dynamic CIC channel characteristics and influencing factors. Section 4 investigates path loss, shadowing, multipath, and Doppler shift. Section 5 presents the composite fading dynamic channel model and analyzes its bit error rate. Section 6 discusses the limitations of the ex vivo experiments and future work. Section 7 concludes the paper.

## 2. Proposed Approaches for Dynamic Channel Measurement

The aim of this approach is not to fully replicate the in vivo physiological environment, but to construct a repeatable and controllable ex vivo platform to capture the true and accurate dynamic channel characteristics. Measurements under such well-controlled conditions provide an indispensable foundation for subsequent in vivo research.

The conventional CIC channel measurements rely on vector network analyzers (VNAs) or radio-frequency (RF) signal generators combined with spectrum analyzers [15,16,19].

These instruments are fundamentally designed for the frequency-domain response of linear time-invariant (LTI) systems and exhibit critical limitations when applied to a beating heart: (1) the measured dynamic characteristic is merely a composite of data from different cardiac phases; (2) the instrument connected to the circuit board is grounded to the earth and the battery-powered transceivers are relatively large in size and cause coupling between them, which introduces uncontrollable measurement errors [20,21]; and (3) the acquisition cannot be triggered synchronously with the cardiac cycle. Therefore, existing studies have captured only discrete samplings of the static response at different time points, rather than the true dynamic channel fading envelope.

**Figure 2:**
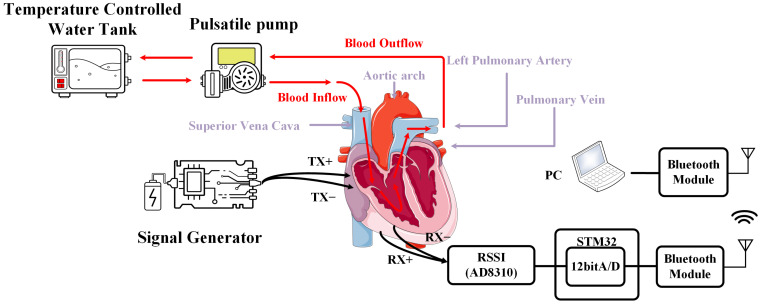
Principle schematic of dynamic channel measurement for CIC.

To address these issues, we develop a miniaturized, battery-powered, continuous-acquisition physical simulation and measurement device specifically designed for dynamic CIC channels. For the first time, this platform achieves synchronous tracking of amplitude–frequency and amplitude–time characteristics within a complete heartbeat. The principle of the dynamic cardiac channel measurement is illustrated in Figure 2. The platform comprises two parts: the physical simulation device and the channel measurement device.

### 2.1. Cardiac Preparation and Perfusion Circuits

**Figure 3:**
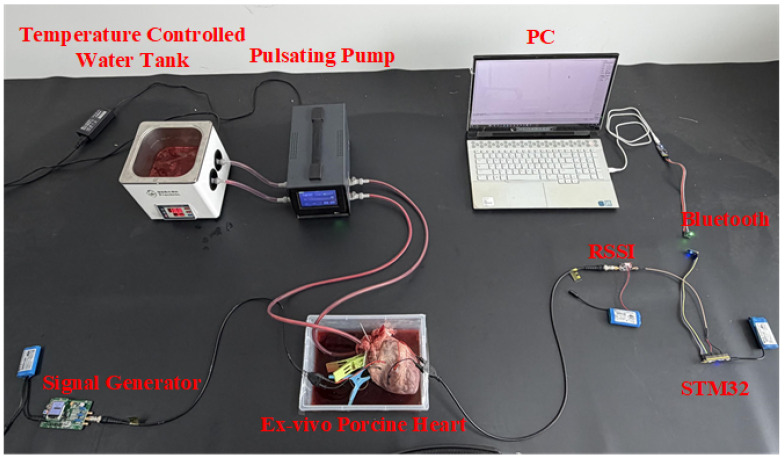
Experimental setup for dynamic channel measurement for CIC. The blue module is a miniaturized lithium battery.

Figure 3 shows the dynamic channel measurement device for ex vivo porcine heart of CIC. The ex vivo porcine hearts are obtained from a local abattoir. Immediately after excision, the hearts are rinsed and perfused with ice-cold, heparinized saline to prevent intravascular clotting and ischemic injury. The time from excision to the start of the experiment is kept under 2 h to preserve myocardial viability.

The physical simulation device consists of a temperature-controlled water tank, a programmable pulsatile pump, and a closed-loop silicone catheter circuit. The tank is filled with sterile anticoagulated porcine blood and kept at 37.0 ± 0.5 °C to simulate the in vivo thermal environment of the human intracardiac cavity. The pulsatile pump is set to 80 beats per minute (bpm) with a stroke volume of 100 mL and a duty cycle of 50%. Blood circulates as follows: water tank → pump → superior vena cava → right atrium → right ventricle → left pulmonary artery → pump → water tank, thereby simulating physiologic blood content, flow rate, and cardiac pulsation. Each heart is allowed to stabilize for 2 min before data acquisition commences. The total experimental duration per heart is limited to within two hours to avoid tissue degradation.

### 2.2. Electrode Design and Placement

Custom needle electrodes are made of stainless steel and designed in a cylindrical shape, with a length of 8 mm and a radius of 5 mm. At the transmitting end, the positive and negative electrodes are vertically inserted into the mid-portion of the right atrium. At the receiving end, the positive and negative electrodes are vertically inserted into the mid-portion of the right ventricle. The distance between positive electrode and negative electrode is 2 cm at both the transmitter and the receiver. The distance between the transmitter and the receiver is 2 cm. The implantation depth from the epicardial surface is 8 mm.

**Figure 4:**
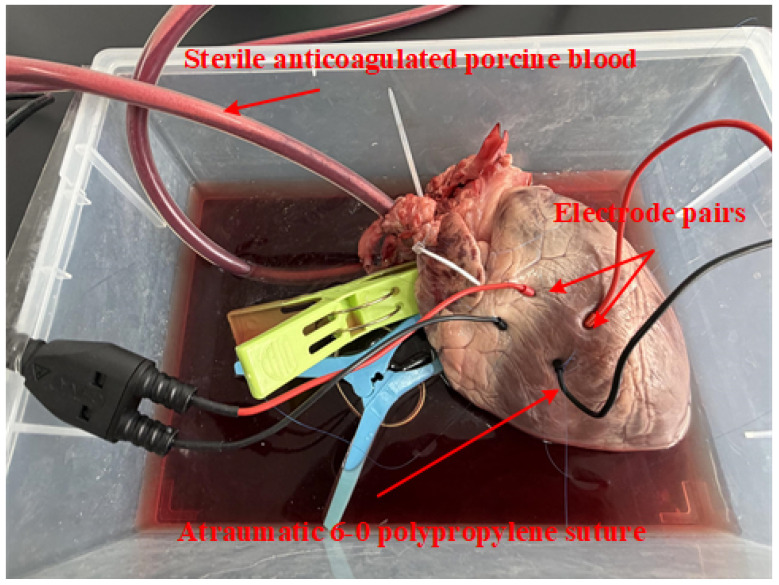
The electrodes are fixed to the surface of the heart through sutures.

To prevent motion-induced displacement, each needle electrode is secured to the epicardial surface with an atraumatic 6-0 polypropylene suture tied around the electrode handle, as shown in Figure 4. This fixation maintains a constant contact area and orientation between the electrodes and the cardiac tissue throughout the cardiac cycle, thereby allowing motion artifacts to be distinguished from genuine channel fading.

### 2.3. Signal Generation and Measurement System

**Figure 5:**
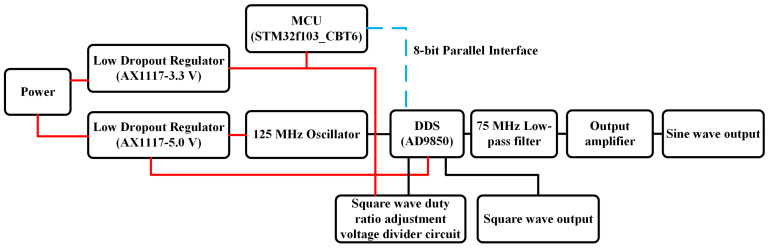
Schematic diagram of the signal generator.

To eliminate instrument-to-ground coupling and inter-device coupling, the entire measurement system is battery-powered and implemented on miniaturized printed circuit boards (PCBs). Figure 5 shows schematic diagram of the signal generator.

A direct digital synthesis (DDS) signal generator is designed using an STM32F103 microcontroller (MCU) (STMicroelectronics, Geneva, Switzerland) and an AD9850 DDS chip (Analog Devices, Wilmington, MA, USA). A split-rail power supply uses two low-dropout linear regulators (LDOs): an AX1117-3.3V power (AXElite Technology, Shenzhen, China) as the MCU, and an AX1117-5.0V supplies (AXElite Technology, Shenzhen, China) both the 125 MHz active oscillator and the AD9850. The 125 MHz oscillator serves as the reference clock for the AD9850, connected to its CLK pin. The MCU interfaces with the AD9850 via an 8-bit parallel bus to configure the frequency tuning word, phase offset, and output amplitude. The AD9850 produces a square-wave output (duty cycle adjustable via a resistive voltage divider) and a raw analog output. The analog output is filtered through a 75 MHz low-pass filter to suppress harmonic components and then amplified to deliver a high-quality sine wave.

A battery-powered received signal strength indicator (RSSI) module captures the transmitted power. Its analog voltage output is digitized by the 10-bit analog-to-digital converter (ADC) of an STM32 microcontroller (STMicroelectronics, Geneva, Switzerland) at a sampling rate of 200 Hz. This sampling rate is approximately 200 times the resting heart rate and satisfies the Nyquist sampling criterion, ensuring that dynamic variations within each cardiac cycle are fully captured without aliasing or information loss. The STM32 streams the digitized data via Bluetooth to a PC for real-time monitoring and processing.

Each experimental condition is recorded continuously for 120 s, capturing at least 100 complete cardiac cycles. All subsequent channel gain calculations and statistical analyses are based on this continuous dataset. Sampling parameters and post-processing steps are kept identical across all measurement setups to enable fair comparison between the proposed DDS-RSSI method and conventional instruments.

### 2.4. Specific Measurement Protocols

All measurements are performed under the stabilized perfusion conditions. The following measurement setup are executed sequentially on each heart:

#### 2.4.1. Amplitude–Frequency and Amplitude–Time Characterization

The DDS signal generator generates a sinusoidal signal with a peak-to-peak value of 1 V. The frequency is set from 100 kHz to 1 MHz and the step is 100 kHz. At each frequency, continuous 120 s amplitude–time waveforms are recorded.

#### 2.4.2. Heart-Rate Variation

To evaluate the effect of cardiac cycle duration on channel gain, the pulsatile pump rate is swept across 40–120 bpm and the step is 10 bpm while stroke volume is 100 mL.

#### 2.4.3. Flow-Rate Variation

To isolate the influence of blood volume and velocity, the pump stroke volume is adjusted from 40 mL to 100 mL in 10 mL steps, while the heart rate is kept constant at 80 bpm. The formula for calculating the flow rate is as follows:

(1) $$Flow\;Rate=\frac{Stroke\;Volume}{\frac{1}{Heart\;Rate}\times Duty\;Cycle}$$

where *Flow Rate* is average volumetric flow rate in mL/min, *Stroke Volume* is volume of blood ejected per pump stroke in mL, *Heart Rate* is pump rate in beats per minute and *Duty Cycle* is the fraction of the cardiac cycle during which the pump is actively delivering flow (dimensionless, 0–1).

#### 2.4.4. Fixed Versus Free Electrode Comparison

Two configurations are tested: (i) needle electrode secured to the epicardial surface with an atraumatic 6-0 polypropylene suture tied around the electrode handle; (ii) identical electrodes inserted to the same depth but without suture fixation, allowing relative motion between the electrode and the myocardium. This comparison quantifies the motion artifact separately from true tissue-channel fading.

#### 2.4.5. Path Loss Versus Distance

To characterize the spatial attenuation, the inter-dipole transmission distance is varied from 2 cm to 10 cm in 2 cm steps, while the intra-dipole spacing remains fixed at 2 cm.

#### 2.4.6. Impulse Response/BER Simulation Input

To estimate the channel impulse response and provide measured waveforms for bit-error-rate (BER) simulation, the transmitter is configured to generate a unipolar baseband pulse train with an amplitude of 5 V, a pulse width of 2 µs, and a repetition period of 10 µs [2,22,23].

## 3. Dynamic Channel Characteristic Analysis of CIC

The dynamic characteristics of the CIC channel are quantified through the following metrics: (1) Receiving voltage $V_{rx}$: The peak-to-peak voltage measured at the receiving electrode pair at each time instant. (2) Received power: Converted from $V_{rx}$ assuming a 50 Ω termination impedance of the measurement system. (3) Channel gain: Defined as the ratio of received power to transmitted power in decibels. This captures the combined effect of tissue attenuation, electrode coupling, and geometric spreading. (4) Path Loss: Path loss is defined as the ratio of the output power at the transmitting end to the input power at the receiving end, usually expressed in decibels. It quantifies the total energy attenuation experienced by the signal as it propagates through the transmission medium from the transmitting end to the receiving end. (5) Dynamic Fading Envelope: The time-varying component of the gain after removing the distance-dependent mean path loss. This envelope is used to extract the shadow-fading distribution. (6) Shadow Fading Standard Deviation ($\sigma_{dB}$): The standard deviation of the gain fluctuations around the mean path-loss model, quantifying the severity of dynamic fading induced by cardiac mechanical motion.

### 3.1. Amplitude–Frequency Characteristic

The signal generator produces a $1V_{pp}$ sine-wave signal with a frequency range of 100 kHz to 1 MHz. The signal passes through the cardiac tissue, and the RSSI input terminal detects the power value of the receiving end. The output terminal of RSSI is the voltage value displayed on the PC, which is the output voltage of RSSI.

**Figure 6:**
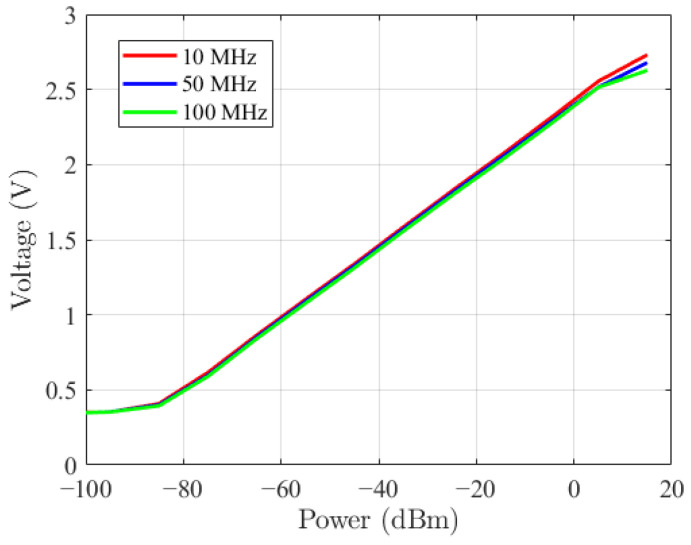
The relationship between the RSSI input power and the RSSI output voltage.

The AD8310 (Analog Devices, Inc., Wilmington, MA, USA) is a logarithmic detector. It detects the radio-frequency energy at the input end, which is the average power. In the application, the input end will match an impedance of 50 Ω. The power value at the receiving end has a linear relationship with the voltage value displayed on the PC, as shown in Figure 6. The formula is as follows:

(2) $$P_{rx}=41.4256\times V_{output}-100.5878$$

$P_{rx}$ is the received power of RSSI, and $V_{output}$ is the voltage value displayed on the computer. This linear relationship is the result shown in Figure 6.

However, the received power $P_{rx}$ is not the channel gain. It needs to be subtracted by the inherent losses such as those of the coaxial cable.

It is necessary to directly connect the electrodes at the transmitting end and the receiving end to obtain the corresponding voltage $V_{0}$ (displayed on the PC). Based on the aforementioned formula, calculate the input power $V_{0}$ when the transmitting end and the receiving end are directly connected.

(3) $$P_{0}=41.4256\times V_{0}-100.5878$$

The channel gain, which is the transmission attenuation in decibels solely caused by the pig heart tissue, is:

(4) $$\mathrm{ChannelGain}\left(dB\right)=P_{rx}\left(dBm\right)-P_{0}\left(dBm\right)$$

**Figure 7:**
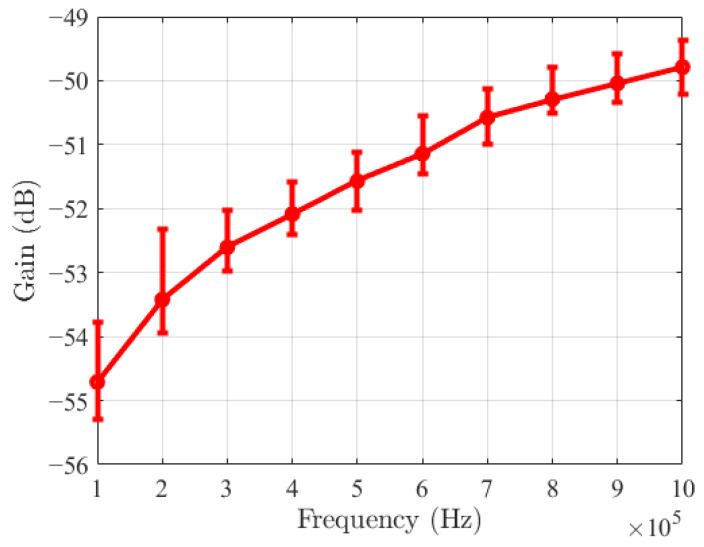
Amplitude–frequency characteristic curve and fluctuation range for CIC.

Figure 7 shows the dynamic CIC channel amplitude–frequency characteristics. The channel gain of CIC exhibits capacitive coupling characteristics. The average channel gain of CIC increases with the increase in frequency, and the channel gain increases from −54.7 dB to −49.8 dB from 100 kHz to 1 MHz.

The vertical interval at each frequency point is the overall change range of channel gain within one heartbeat cycle. Within one heartbeat cycle, the minimum fluctuation of channel gain is 0.7 dB at 700 kHz, and the maximum is 1.62 dB at 200 kHz. The fluctuation range at each frequency point is not significantly different.

### 3.2. Amplitude–Time Characteristic

To measure the dynamic changes of channel gain within a heartbeat cycle, we use a signal generator to output a sine excitation signal at a specific frequency. The dynamic process of the channel variation is shown in Supplementary Video S1.

**Figure 8:**
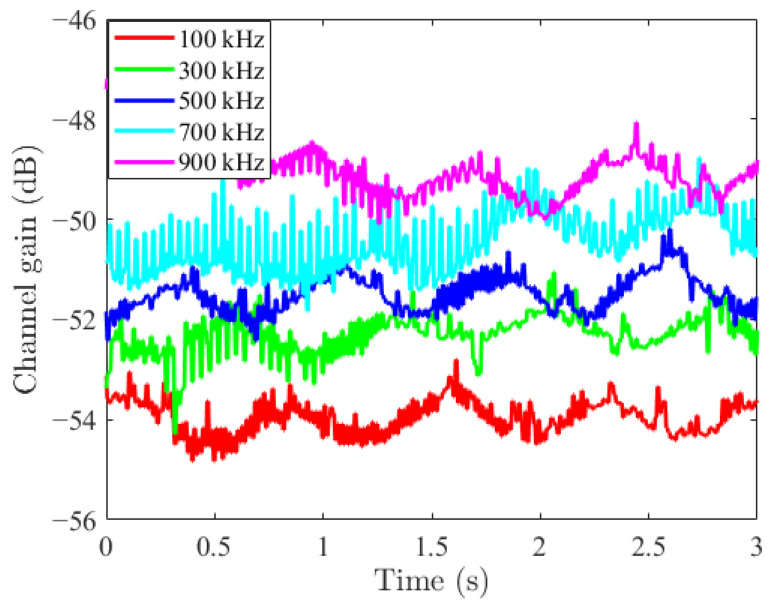
Amplitude–time characteristic curve and fluctuation for CIC.

Figure 8 illustrates the variation in channel gain across the cardiac cycle at different frequencies. The channel gain at low frequencies is less than that at high frequencies. Curves of all frequencies show obvious periodic fluctuations, indicating that the channel gain of CIC varies periodically with the heartbeat movement. The fluctuation is mainly due to periodic length changes in the myocardium induced by the heartbeat.

The fluctuations at low frequencies are larger (1.5 dB at 100 kHz), while they become smaller at higher frequencies (0.8 dB at 900 kHz). This is because the wavelength of low-frequency signals is longer, allowing them to penetrate deeper into the heart. The current covers a larger volume of myocardial tissue, has a longer transmission path, is more affected by overall deformation, and has a high sensitivity to tissue deformation. While high-frequency signals have a shallower penetration depth, they mainly propagate along the surface of the heart, have a shorter path, and are less affected by overall heart deformation.

### 3.3. Analysis of Influencing Factors on Channel Characteristics

Considering that the experimental results may be affected by various factors, we investigate the influence of different measuring instruments, heart rate, flow rate and motion artifacts on the channel characteristics.

#### 3.3.1. Instrument Difference

**Figure 9:**
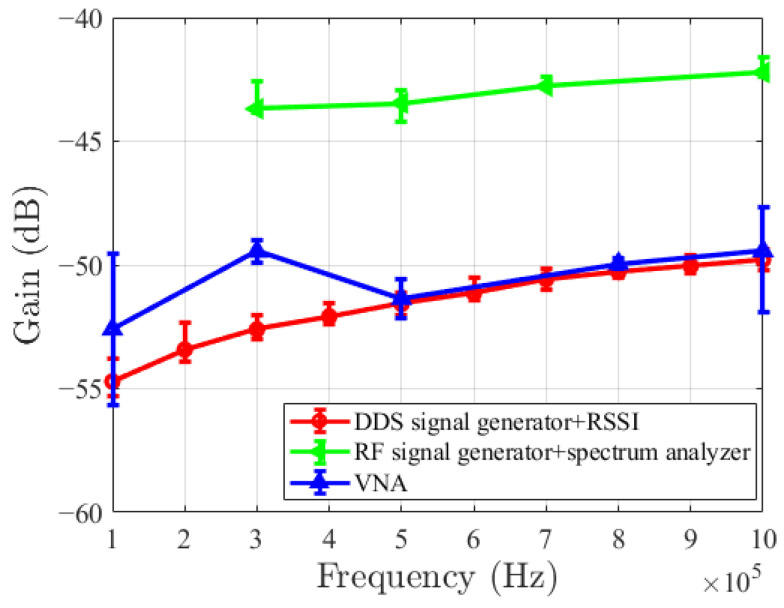
The CIC channel gain with different experimental instruments. Red curve: DDS signal generator + RSSI. Green curve: The RF signal generator typically limits the minimum output frequency to 250 kHz. Blue curve: The VNA shows significant noise below 1 MHz.

To verify the validity and accuracy of the measurement data, we conduct three sets of comparative experiments. Figure 9 presents the channel gains obtained from the three different experimental setups. The red curve represents the results using the DDS signal generator and RSSI method proposed in this paper. The green curve corresponds to measurements taken with an RF signal generator and spectrum analyzer. The blue curve shows the data acquired using the vector network analyzer.

The results show that the channel gain obtained using the DDS signal generator and RSSI ranges from −54.7 dB to −49.8 dB over the frequency range of 100 kHz to 1 MHz.

The channel gain obtained using the RF signal generator and spectrum analyzer ranges from −43.69 dB to −42.22 dB over the frequency range of 300 kHz to 1 MHz, increasing 1.47 dB. The maximum fluctuation amplitude within one cardiac cycle is 1.3 dB, and the minimum fluctuation amplitude is 0.43 dB, with a difference of 0.87 dB.

The channel gain measured using the vector network analyzer range from −52.60 dB to −49.43 dB over the frequency range of 100 kHz to 1 MHz, increasing of 3.17 dB. The maximum fluctuation amplitude within one cardiac cycle is 6.13 dB, and the minimum fluctuation amplitude is 0.4 dB, with a difference of 5.73 dB.

The channel gain trends obtained through these three methods are consistent, and they exhibit capacitive coupling characteristics. The key differences lie in the performance and limitations of each instrument. The experimental configuration using DDS signal generator combined with RSSI module offers high time resolution and stable, continuous data acquisition, making it well-suited for capturing dynamic channel variations within cardiac cycle.

In contrast, measurements using RF signal generator and spectrum analyzer are limited by lower sampling frequency. The RF signal generator has a minimum output frequency of 250 kHz. The substantial physical dimensions of the RF signal generator and spectrum analyzer introduce significant parasitic capacitance. This leads to an overestimation of the measured channel gain, as the signal bypasses the tissue medium through unintended parasitic paths.

The vector network analyzer provides comprehensive frequency-domain data but exhibits higher noise levels at low frequencies. This results in significant data fluctuations and distortion within 100 kHz to 300 kHz, compromising measurement accuracy in the lower band.

Therefore, the proposed DDS + RSSI system, by cutting off parasitic capacitance and using continuous sampling instead of sweep mode, has for the first time achieved point-by-point tracking of channel gain within a continuous heartbeat.

#### 3.3.2. Heart Rate

**Figure 10:**
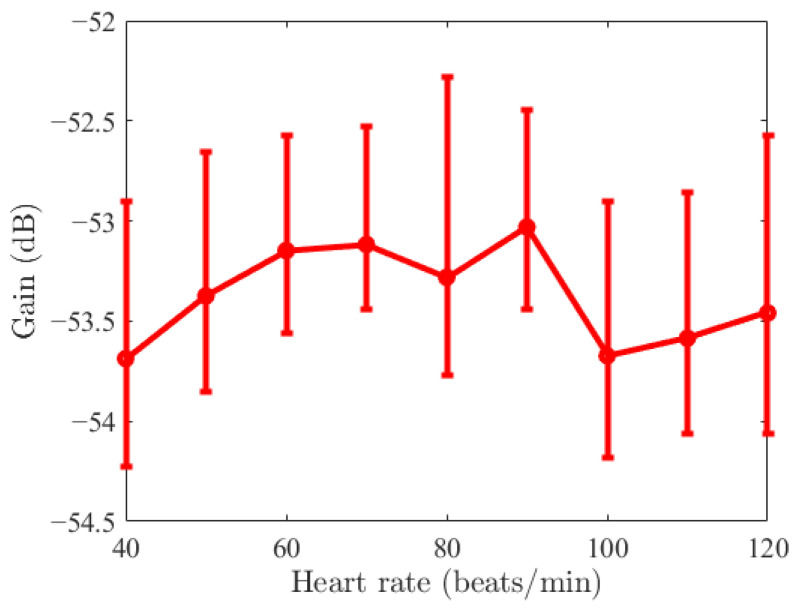
Heart rate on CIC channel characteristics.

Taking into account that heart rates vary among individuals, we investigate the impact of different heart rates on the channel characteristics. Figure 10 shows the influence of heart rate on channel gain. When the heart rate increases from 40 BPM to 120 BPM, the average channel gain varies within the range of −53.7 dB to −53 dB, with a difference of 0.7 dB.

Table 1 shows the average gain and fluctuation range for CIC dynamic channels under varying heart rates. Within a single cardiac cycle, the maximum fluctuation of channel gain is 1.49 dB at 120 beats/min, and the minimum fluctuation of channel gain is 0.9 dB at 70 beats/min.

These results show that the channel remains generally stable under normal conditions. However, in extreme conditions, channel compensation mechanisms for the CIC system may be necessary.

#### 3.3.3. Flow Rate

In a resting state, the cardiac output of a healthy adult heart ranges from 67 to 133 mL/s. However, cardiovascular conditions such as valvular regurgitation, atrial septal defect, and coronary artery disease can disrupt normal hemodynamics, resulting in either excessive or insufficient blood flow. Therefore, this study investigates how variations in cardiac pumping volume affect the channel characteristics.

**Figure 11:**
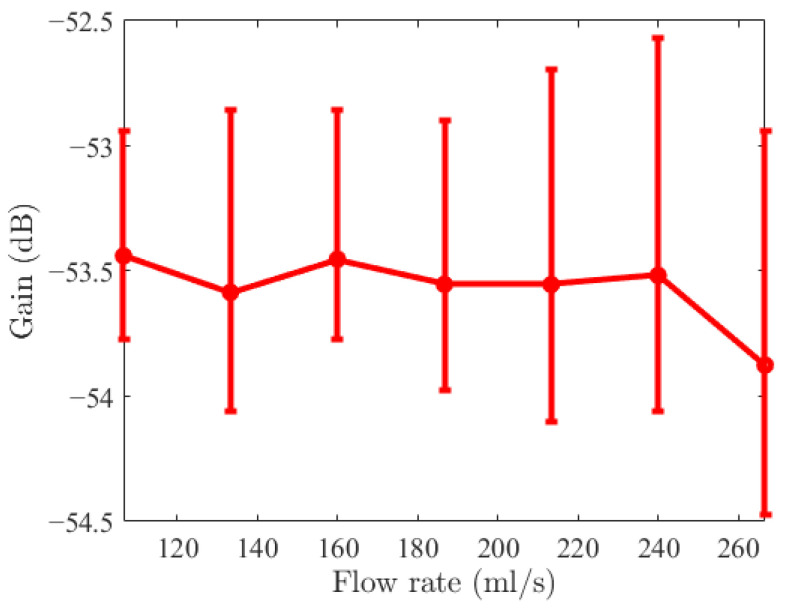
Flow rate on CIC channel characteristics.

Figure 11 illustrates the influence of flow rate on channel gain. When the blood flow rate changes from 106 mL/s to 266 mL/s, the average channel gain changes from −53.44 dB to −53.88 dB, and the fluctuation range changes from 0.83 dB to 1.53 dB. These experimental results indicate that as blood flow rate increases, the fluctuation range of the channel gain becomes larger, the channel attenuation increases, and the overall channel condition deteriorates. Table 2 presents the average gain and fluctuation range of CIC dynamic channels at different flow rates.

#### 3.3.4. Analysis of Motion Artifacts

To distinguish between intrinsic channel attenuation and motion artifacts, we compare the channel gain measured by free electrodes and fixed electrodes. The free electrode is defined as an electrode vertically inserted into the myocardial tissue without any fixation. The fixed electrode is defined as an electrode vertically inserted into the myocardial tissue and secured to the heart surface with an atraumatic 6-0 polypropylene suture tied around the electrode handle.

**Figure 12:**
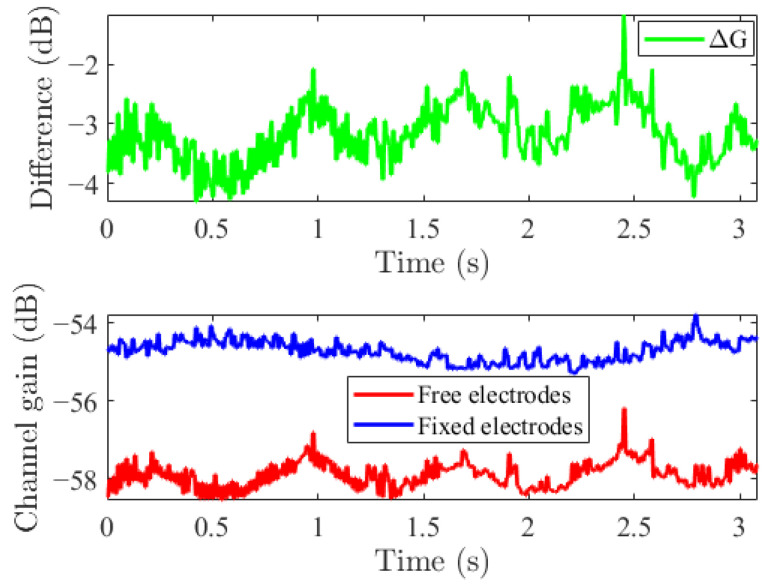
The difference in channel gain measured on the heart surface between the free electrodes and the fixed electrodes.

Figure 12 illustrates the channel gain fluctuations over a 3 s interval, comparing free and fixed electrodes across several cardiac cycles. The blue curve represents the intrinsic channel response of the fixed-electrode configuration. It exhibits high stability with fluctuations under 1 dB, accurately reflecting true tissue conductivity and geometric path loss while remaining largely unaffected by mechanical heart motion.

In contrast, the red curve (free electrodes) displays significant periodic fluctuations synchronized with the cardiac cycle, with an amplitude of 3–4 dB. The consistently higher gain of the fixed configuration indicates that motion-induced contact-impedance variation introduces substantial additional attenuation.

This motion artifact is quantified by the difference $\Delta G\left(t\right)=G_{\mathrm{free}}\left(t\right)-G_{\mathrm{fixed}}\left(t\right)$ shown as the green curve. ΔG(t) remains negative throughout the cycle, averaging around −3 dB with a variation of roughly 2 dB, which confirms that free electrodes suffer from greater signal loss due to intermittent tissue-electrode contact.

At t≈0.5s, 1.3s, 2.1s, and 2.9s, the red curve exhibits distinct troughs. These time points correspond to the cardiac systolic phases. During this interval, the cardiac muscle contracts vigorously, mechanically pushing the free electrode away. This action causes the contact impedance to increase instantly, resulting in the most severe signal attenuation. Thus, the channel gain drops to its lowest point, representing the moment when motion artifacts are the most severe.

Conversely, at t≈1.0s, 1.8s, and 2.6s, the red curve reaches local peaks. These correspond to the cardiac diastolic phases. As the cardiac muscle relaxes, the free electrode rebounds, re-establishing good contact with the tissue. At this time, the contact impedance decreases, allowing the signal to recover. Notably, the gain value of the free electrode (red curve) becomes very close to that of the fixed electrode (blue curve). This indicates that during the diastolic phase, the influence of motion artifacts is minimal, and the measured signal is closest to the true channel gain.

The results confirm that the severe fluctuations in the free electrode are primarily motion artifacts rather than intrinsic channel changes. Therefore, secure mechanical fixation is critical for suppressing these artifacts in intra-body communication.

## 4. Analysis of Channel Fading and Propagation Effects for CIC

### 4.1. Path Loss

Since the pacemaker is installed at different positions in the heart according to the patient’s specific condition, the path loss reduces the average signal energy. We model path loss as a function of distance, with the average path loss following a power-law relationship proportional to the Nth power of distance [24,25].

The transmission distance is varied from 2 cm to 10 cm, and the step is 2 cm with seven measurements recorded at each distance. The fitting curve is derived using the average value at each point to minimize errors arising from measurement variability and experimental fluctuations.

Since the distance between the transmitter and the receiver is very short at the centimeter level, the system is completely within the reactive near-field region. Signals propagate through conductive current and displacement current rather than electromagnetic radiation. The electric field distribution is close to the quasi-static electric field.

**Figure 13:**
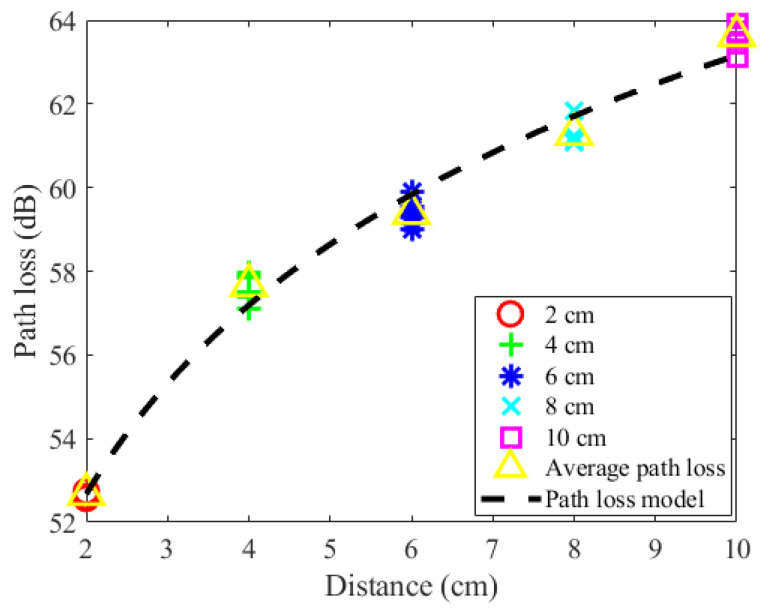
Average path loss and model for CIC.

From the measurement results in Figure 13, it can be concluded that the voltage attenuation might be slower than $1/d^{2}$. The increase in path loss gradually slows down as the distance increases. The path loss model uses a logarithmic model:

(5) $$PL_{dB}\left(d\right)=PL_{0}+\gamma \cdot log_{10}\left(d/d_{0}\right)$$

where $PL_{dB}\left(d\right)$ is the path loss in dB, $PL_{0}$ is the path loss at $d_{0}$, and γ is the path loss index.

Figure 13 shows the path loss results and near-field coupling path loss model. As the channel length increases from 2 cm to 10 cm, the path loss increases by 10.96 dB. When γ = 15.012, the root mean square error of the model is 0.455. The fitted path loss exponent n=γ/10≈1.5 indicates that the signal attenuation is weaker than in free space (*n* = 2) and significantly weaker than the theoretical electric dipole field in a conductive medium (*n* = 4). This path loss is consistent with quasi-static field behavior in biological tissues at low frequencies, where current diffusion dominates over radiative effects.

### 4.2. Shadow Fading

In the scenario where both the transmitter and the receiver are located on the beating heart, the shadowing effect is caused by the large-scale signal attenuation resulting from the dynamic occlusion and absorption of the signal due to the deformation of the myocardium, the flow of blood, and the periodic displacement of the surrounding tissues. Therefore, we analyze the dynamic shadowing effect of cardiac physiological modulation.

At the TX side, the signal generator outputs a pulse signal with an amplitude of 5 V, a frequency of 100 kHz, a pulse width of 2 µs, and a duty cycle of 20%. At the RX side, the signal is detected by the RSSI circuit and sampled at 200 Hz by a 10-bit analog-to-digital converter (ADC) of STM32. The acquired data is sent to the PC through the Bluetooth module of STM32. The heart cycle of the heart is 0.8 s, and a sampling rate of 200 Hz can meet the requirements of channel measurement [26,27].

**Figure 14:**
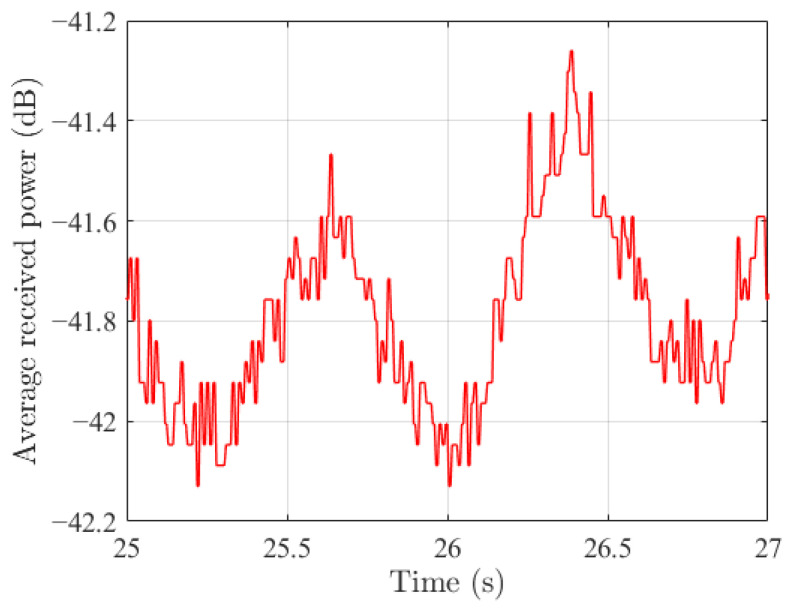
Snapshots of power gain ratio. The average received power data from the 25th second to the 27th second.

Figure 14 shows the fluctuation range of power gain over heartbeat cycles. The RSSI records a total of 138.6 s of continuous data. The first 20 s is discarded to allow for hemodynamic stabilization following catheter connection and pulsatile pump priming, leaving a 100 s analysis window (20 s to 120 s).

A moving-average filter is applied with a window length of one cardiac cycle ($T_{c}=0.75$ s at 80 bpm) and a step size of $T_{c}/4$ (0.19 s), yielding M=523 local-mean samples:

(6) $$M=\left\lfloor \frac{T_{\mathrm{analysis}\;}-T_{\mathrm{window}\;}}{T_{\mathrm{step}\;}}\right\rfloor +1$$

To ensure statistical independence, the normalized autocorrelation function (ACF) of $\bar{G}\left[m\right]$ is computed as:

(7) $$\rho \left[k\right]=\frac{\sum_{m=1}^{M-k}\left(\bar{G}\left[m\right]-\mu_{\bar{G}}\right)\left(\bar{G}\left[m+k\right]-\mu_{\bar{G}}\right)}{\sum_{m=1}^{M}{\left(\bar{G}\left[m\right]-\mu_{\bar{G}}\right)}^{2}},$$

where *k* denotes the lag index and $\mu_{\bar{G}}$ is the sample mean.

The ACF decays below the 1/e threshold (ρ=0.368) at lag $k_{0}=6$, corresponding to a decorrelation time $\tau_{\mathrm{decorr}}=k_{0}\times 0.19\;s\approx 1.14$ s. The shadow fading sequence is therefore downsampled by retaining every 6th sample, resulting in N≈88 independent samples per heart.

(8) $$N=\left\lfloor \frac{M-1}{k_{0}}\right\rfloor +1$$

Across all 5 hearts, the total independent sample size is $N_{\mathrm{total}}=440$.

The shadow fading of the *n*-th independent sample is defined as:

(9) $$S\left[n\right]=\bar{G}\left[n\cdot k_{0}\right]-{\hat{G}}_{\mathrm{PL}}\left(d=2\;\mathrm{cm}\right),\;n=1,2,\ldots ,N_{\mathrm{total}}$$

The empirical distribution of the pooled *S* is tested for normality in the dB domain. The parameters of the dB-domain Gaussian distribution are estimated using maximum likelihood estimation (MLE). The goodness-of-fit is assessed by the Anderson–Darling (A–D) test against the null hypothesis of a Gaussian distribution [26,28]. The A–D statistic does not reject normality, confirming that the shadow fading in the ex vivo CIC channel is well described by a log-normal envelope in the linear domain.

**Figure 15:**
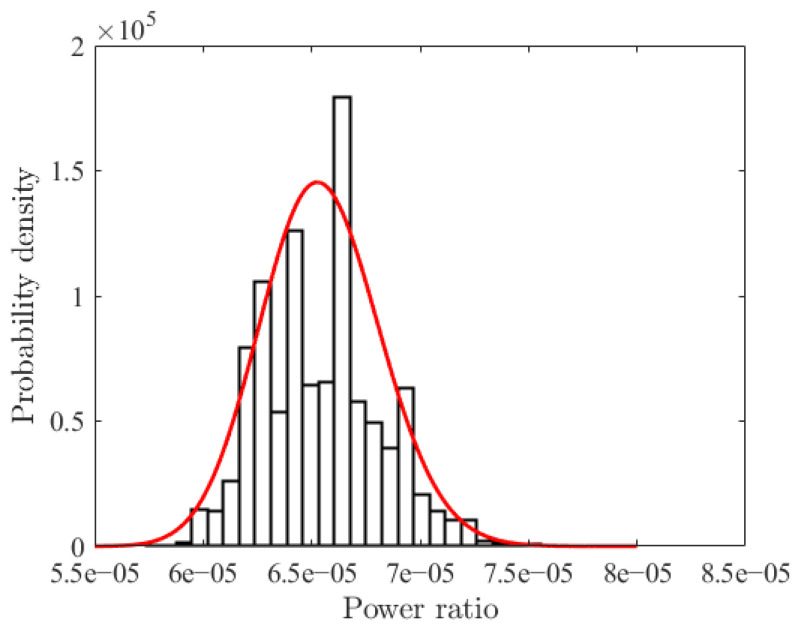
Histogram of the Power Ratio with the fitted Lognormal Probability Density Function.

The fitted parameters of the dB-domain Gaussian distribution are:

- Mean: $\mu_{dB}=-1.4\times 10^{-15}$ dB;
- Standard deviation: $\sigma_{dB}=0.1842$ dB;

Figure 15 shows the histogram of the received power ratio fitted with a log-normal probability density function (PDF). The standard deviation ($\sigma_{S}<1$ dB) quantitatively confirms that the shadow fading in the low-frequency near-field CIC channel is exceptionally weak.

### 4.3. Multipath Fading

Multipath fading is caused by the distribution of reflectors and scatterers along the signal transmission path. The multipath effect requires the existence of multiple distinguishable propagation paths. There is a significant time delay difference between each path. The phase of each path signal is random or time-varying, resulting in fluctuations at the receiving end due to coherent superposition of the signals.

**Figure 16:**
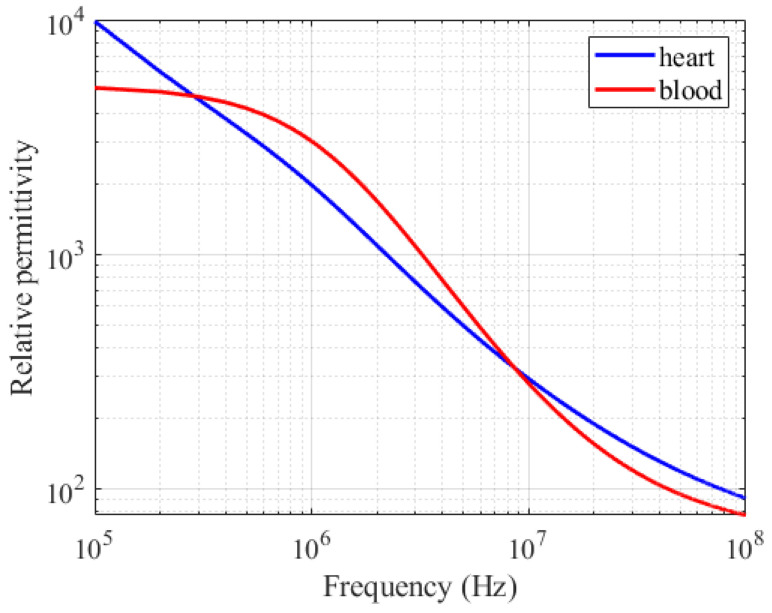
Relative permittivity of blood and heart from 100 kHz to 100 MHz.

**Figure 17:**
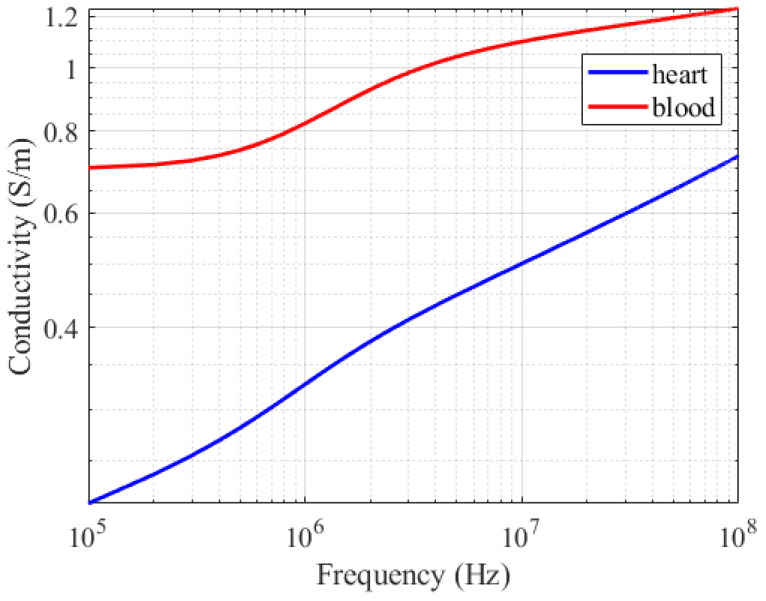
Conductivity of blood and heart from 100 kHz to 100 MHz.

According to the fourth-order cole–cole dispersion Formulas (10)–(12) [29], the relative permittivity and conductivity of the heart and blood are shown in the Figure 16 and Figure 17.

(10) $$\begin{array}{c} \hat{\varepsilon }\left(\omega \right)=\varepsilon_{\infty }+\sum_{n}\frac{\Delta \varepsilon_{n}}{1+{\left(j\omega \tau_{n}\right)}^{\left(1-\alpha_{n}\right)}}+\frac{\sigma_{i}}{j\omega \varepsilon_{0}} \end{array}$$

(11) $$\begin{array}{c} \varepsilon^{\prime }=\mathrm{real}\left(\hat{\varepsilon }\right) \end{array}$$

(12) $$\begin{array}{c} \sigma =-\mathrm{imag}\left(\hat{\varepsilon }\right)\cdot \omega \cdot \varepsilon_{0} \end{array}$$

The high conductivity of the myocardial tissue limits the penetration depth of electromagnetic waves. There are no obvious strong reflection interfaces within the tissue. The dielectric properties of the myocardial tissue gradually change rather than abruptly change, and the reflection coefficient is extremely small. Even if there is a weak reflection, it will be rapidly attenuated by high absorption and cannot form a measurable secondary path.

When the wavelength of the electromagnetic wave is much larger than the size of the heart, the electromagnetic field is in the quasi-static near-field region. Energy is transferred through both conduction current and displacement current. The displacement current originates from the dielectric polarization response of the tissue under the external electric field.

Suppose there are two paths, the length difference ΔL = 0.01 m, the speed of electromagnetic waves in cardiac tissue $v_{p}\approx c/\sqrt{\varepsilon^{\prime }}$ = $3\times 10^{6}$ m/s, the time delay difference $\Delta \tau =\Delta L/v_{p}\approx 3.3\times 10^{-9}\;s=3.3\;\mathrm{ns}$, the corresponding coherent bandwidth $B_{c}\approx 1/\left(5\Delta \tau \right)\approx 60$ MHz, and the channel bandwidth is less than 1 MHz. Δτ≪1/B. All the paths are completely in-phase-superimposed at the receiving end, resulting in an enhanced single-path signal with no frequency selectivity and no fading.

### 4.4. Analysis of Doppler Effect

Since TX is located in the right atrium and RX in the right ventricle, during the contraction of the heart, the movements of different parts are not synchronized: the atrium contracts first, followed by the ventricle; the apex of the heart swings with a large amplitude, while the base has a smaller amplitude. The distance between the two points will change periodically.

The physical mechanism of channel fading caused by the relative motion between the transmitter and the receiver due to the beating of the heart is the Doppler effect.

The rate of channel change is represented by the Doppler shift $f_{d}$:

(13) $$f_{d}=\frac{v_{\mathrm{rel}}}{c_{\mathrm{med}}}f_{c}$$

where $f_{d}$ is the Doppler shift in Hz, $v_{rel}$ is the relative velocity component (radial velocity) between the transmitters and receivers along the direction of signal propagation, $c_{med}$ is the velocity of wave propagation in the medium, and $f_{c}$ is carrier frequency in Hz.

Suppose the variation range of the transmission and reception distance Δd=0.01m, the heart rate is 1 Hz, approximately representing simple harmonic motion, the maximum velocity $v_{\max}=2\pi f_{\mathrm{heart}}\cdot \Delta d\approx 0.062\;m/s$, and the maximum Doppler frequency shift $f_{d}=\frac{v_{\max}}{v_{p}}f_{c}\approx 0.002\;\mathrm{Hz}$. The bandwidth of the CIC signal ranges from several kHz to several MHz. It is simply unable to distinguish such a tiny frequency shift, and the Doppler effect is extremely weak.

Therefore, the Doppler spread is negligible. The channel variations caused by heartbeats manifest primarily as slow fluctuations in path loss rather than fast fading. Consequently, the CIC channel can be modeled as a slow-fading channel without significant Doppler effects.

## 5. Analyze of Composite Fading Dynamic Channel Model for CIC

### 5.1. Proposed Composite Fading Dynamic Channel Model of CIC

Based on the aforementioned measured data, we propose a composite fading dynamic channel model of CIC. The parameters of this model are obtained by fitting with the measured data, indicating that it can effectively describe the statistical characteristics of the measured channel. The formula for received power gain is:

(14) $$P_{r}\left[m\right]={\left(10^{-\frac{PL_{0}+\gamma log_{10}\left(d/d_{0}\right)}{20}}\cdot 10^{\frac{Z\left(mT_{s}\right)}{20}}\right)}^{2}\cdot P_{t}+P_{n}$$

where $P_{r}\left[m\right]$ is the received signal at the m-th sampling moment, $T_{s}$ is the sampling period, $P_{t}$ is transmitted power, and $P_{n}$ is noise power.

(15) $$X_{shadow}\left(mT_{s}\right)=10^{Z(mT_{s})/20},Z\left(mT_{s}\right)∼N\left(\mu_{shadow},\sigma_{shadow}^{2}\right)$$

where $X_{shadow}\left(mT_{s}\right)$ is a shadow fading factor in the linear domain, while $Z(mT_{s})$ is the shadow fading in dB and follows a normal distribution.

Convert the above formula into dB form:

(16) $$P_{r}^{\left(\mathrm{dB}\right)}\left[m\right]=P_{t}^{\left(\mathrm{dB}\right)}-\left(PL_{0}+\gamma \log_{10}\left(\frac{d}{d_{0}}\right)\right)+Z\left(mT_{s}\right)$$

### 5.2. BER Analysis for Dynamic Channel Model of CIC

Based on the channel characterization results above, we further analyze the BER performance of the composite fading dynamic channel model for CIC. The simulation adopts the following measured parameters: distance-dependent path loss with exponent n=1.5, and log-normal shadowing with negligible mean bias $\mu_{dB}=-1.4\times 10^{-15}$ dB and standard deviation $\sigma_{dB}=0.1842$ dB. The evaluated frequency configurations include a unipolar baseband pulse, 100 kHz, and 1 MHz carrier frequencies.

According to the IEEE 802.15.6 protocol standard [30], the transmitted signal is configured as a unipolar baseband pulse with an amplitude of 5 V, a pulse width of 2 µs, a cycle of 10 µs, and a data rate of 100 kbps. To ensure a fair comparison, we adopt the normalized signal-to-noise ratio $E_{b}/N_{0}$.

**Figure 18:**
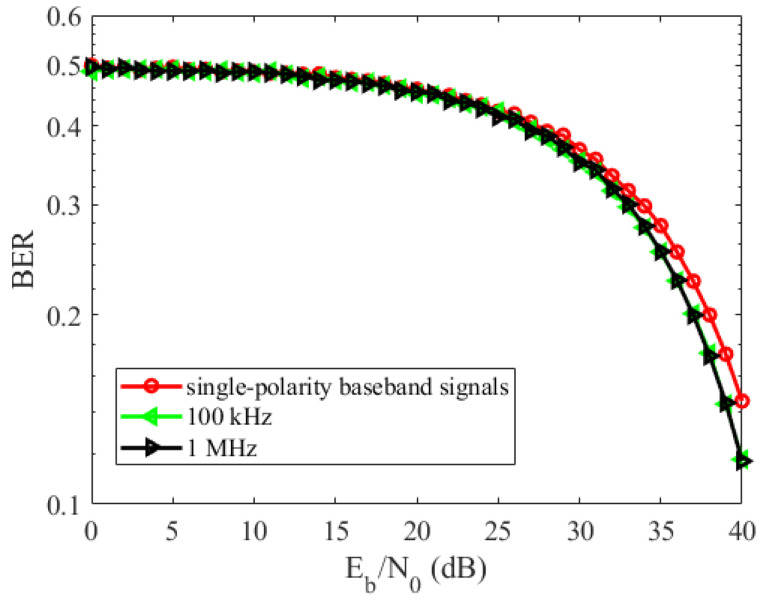
Simulation of BER for baseband signal and OOK modulation with carrier frequencies of 100 kHz and 1 MHz. The simulation is based on the channel parameters measured in this paper.

Figure 18 shows the BER performance of the single-polarity baseband signal, 100 kHz, and 1 MHz OOK modulation under the CIC channel. All curves exhibit a slow decreasing trend as $E_{b}/N_{0}$ increases. In the low-SNR regime, the BER remains near 0.5, indicating that the channel is severely fading-limited.

Although $E_{b}/N_{0}=40$ dB serves as an upper-boundary reference for trend analysis and may be challenging to achieve in power-constrained leadless pacemaker hardware, the BER remains approximately 0.14. This confirms that the CIC channel suffers from severe composite fading, where the joint effect of path loss and shadowing introduces significant attenuation that dominates over thermal noise, making BER reduction difficult even at high SNR. Notably, the measured shadow fading is relatively weak ($\sigma_{dB}=0.1842$ dB), indicating that the dominant impairment is the large-scale path loss rather than dynamic shadowing.

At $E_{b}/N_{0}=40$ dB, the OOK-modulated carrier signals (100 kHz and 1 MHz) achieve a BER roughly 0.03 lower than that of the single-polarity baseband signal, while the two carrier frequencies perform nearly identically. This suggests that, under the present uncoded OOK scheme, merely adjusting the carrier frequency within this range offers limited benefit for BER improvement.

According to $E_{b}/E_{0}$ = 40 dB and *SNR* = $10^{{\mathrm{SNR}}_{\mathrm{dB}}/10}$, we substitute *B* = $10^{6}$ Hz and *SNR* = $10^{4}$ into *C* = ${\mathrm{Blog}}_{2}\left(1+\mathrm{SNR}\right)$, and C≈ 13.29 Mbps.

The marked discrepancy between this ideal capacity and the high practical BER observed with simple OOK modulation in a severe path-loss-limited channel underscores the substantial performance degradation caused by tissue attenuation. Bridging this gap in practice would require sophisticated channel coding, interleaving, and equalization strategies.

It should be emphasized that these results are intended solely as indicative trends rather than precise predictions of clinical device performance. The actual BER may vary significantly in specific implementations depending on transceiver design, electrode–tissue interface impedance, and tissue heterogeneity. Furthermore, advanced techniques such as channel coding or modulation schemes could substantially improve the BER [31,32,33,34].

## 6. Discussion

### 6.1. Static vs. Dynamic Channel Characterization

#### 6.1.1. Static and Dynamic Amplitude–Frequency Curves

**Figure 19:**
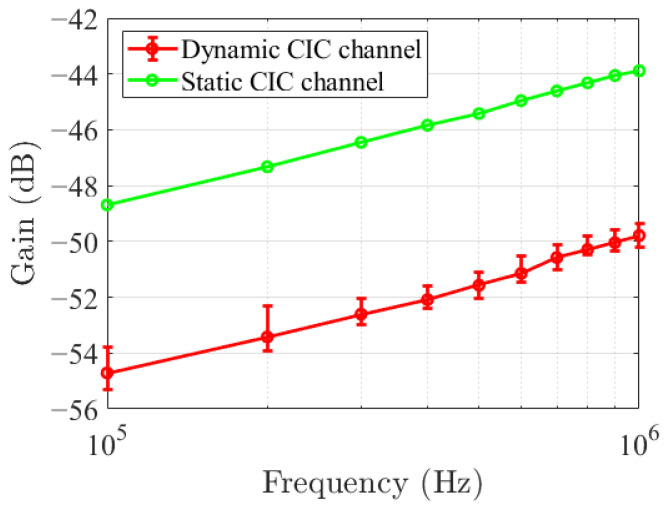
Amplitude–frequency characteristics of the static and dynamic heart.

Figure 19 compares the channel gain versus frequency for the static and dynamic heart at a fixed electrode spacing of 2 cm. Both configurations exhibit the same qualitative trend. The channel gain increases monotonically from 100 kHz to 1 MHz, consistent with the frequency-dependent dielectric dispersion of cardiac muscle.

The static heart exhibits a significantly higher mean gain than the dynamic heart across the entire frequency band. At 100 kHz, the static channel gain is approximately −48.5 dB, whereas the dynamic channel gain is −54.5 dB. At 1 MHz, the gap remains approximately 6 dB. This indicates that cardiac mechanical motion degrades the average channel quality. The mechanism is attributed to the dynamic contact-impedance degradation. Under pulsatile beating, the myocardial wall undergoes cyclic contraction and relaxation, generating micro-displacement and shear stress at the needle–tissue interface. This motion disrupts the stable galvanic contact established in the static state, introducing intermittent micro-gaps and fiber reorientation that elevate the average electrode–tissue impedance. In contrast, the static heart maintains a mechanically quiescent interface where the needle electrodes remain firmly embedded in relaxed tissue, yielding lower contact resistance and higher gain.

Secondly, the frequency-dependent slope is nearly identical between the two models, confirming that the intrinsic tissue dielectric properties are preserved. The static curve rises by approximately 4.7 dB from 100 kHz to 1 MHz, while the dynamic curve rises by approximately 4.7 dB over the same band. The parallelism of the two curves validates that the path-loss exponent n and the frequency-dispersion mechanism are intrinsic to the myocardial tissue and are not altered by the mechanical motion. The 6 dB vertical offset is therefore purely attributable to the time-averaged interface loss induced by heartbeat, not to a change in tissue electromagnetic properties.

Thirdly, the error bars reveal the signature of dynamic fading. The static channel exhibits small, uniform error bars (0.5–1 dB), reflecting the measurement uncertainty and minor tissue heterogeneity in a time-invariant channel. The dynamic channel shows substantially larger error bars (1–2 dB), which directly quantify the shadow fading caused by periodic cardiac contraction. These error bars represent the standard deviation of the gain fluctuations over multiple heartbeat cycles, and their magnitude is consistent with the extracted shadow-fading parameter $\sigma_{dB}$ = 0.1842 dB.

In summary, the static model captures the intrinsic tissue path-loss architecture under idealized mechanical conditions, whereas the dynamic model reveals that cardiac motion introduces both a deterministic average gain penalty of 6 dB due to contact-impedance degradation and a stochastic fading component superimposed on the mean. Both effects are essential for realistic leadless pacemaker link-budget design.

#### 6.1.2. Static and Dynamic Model

The static model is

(17) $$P_{r}^{\left(\mathrm{dB}\right)}=P_{t}^{\left(\mathrm{dB}\right)}-\left(PL_{0}+\gamma \log_{10}\frac{d}{d_{0}}\right)$$

And the dynamic model is The static model is

(18) $$P_{r}^{\left(\mathrm{dB}\right)}\left[m\right]=P_{t}^{\left(\mathrm{dB}\right)}-\left(PL_{0}+\gamma \log_{10}\frac{d}{d_{0}}\right)+Z\left(mT_{s}\right)$$

$Z(mT_{s})$ represents a time-varying random process. The value of m varies at each sampling time, and it describes the fluctuation of power over time within the heartbeat cycle, depicting the changes in electrode–tissue contact impedance caused by the heartbeat and the fluctuations in power resulting from changes in the geometric length of the heart. Most fall within the ±0.37 dB range (with a 95% probability), the mean is close to 0, and the standard deviation is 0.1842 dB.

However, the static model does not have such time-varying terms. It is considered that the received power is a deterministic function of distance and frequency. The received power is deterministic and is solely determined by distance and frequency.

### 6.2. Statistical Analysis

Measurements are performed on 5 independent porcine hearts to assess biological reproducibility. For each heart and each experimental condition, continuous 120 s recordings are acquired, capturing at least 100 complete cardiac cycles per recording. Channel gain is computed from the calibrated RSSI data.

Across all 5 hearts, the inter-heart variability in channel gain is found to be negligibly small: the maximum deviation in mean gain between any two hearts is less than 1 dB across all tested frequencies, heart rates, and flow conditions. This high reproducibility indicates that the ex vivo dynamic CIC channel behavior is dominated by controlled perfusion status and electrode geometry rather than uncontrolled inter-individual biological variability.

**Figure 20:**
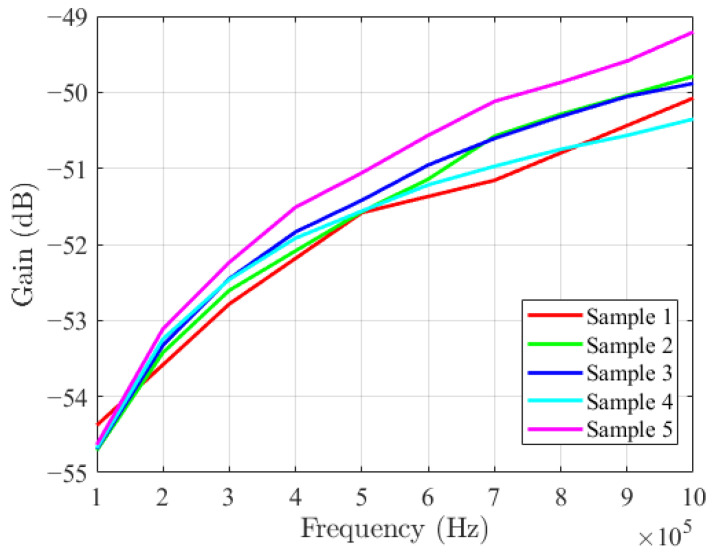
Comparison of amplitude–frequency characteristics across all five porcine hearts.

Figure 20 overlays the amplitude–frequency curves from all five hearts, confirming that the representative traces fall within a narrow inter-heart fluctuation envelope (±1 dB). All quantitative metrics, including path loss exponents, shadow fading variance, and Doppler spread, are calculated based on this representative dataset; the relative deviations of these metrics across the other four hearts are all less than 5%.

### 6.3. Scope of the Conclusions

The conclusions drawn in this study are strictly bounded by the experimental conditions under which the data were acquired. Specifically, the characterization of the CIC channel as an extremely slow-fading channel with negligible multipath and immeasurable Doppler shift applies exclusively to:

(1) Frequency range: 100 kHz to 1 MHz, where the conductive mechanism dominates and the wavelength in tissues is orders of magnitude larger than the inter-electrode distance (2 cm). Under this near-field condition, displacement currents are negligible and the channel is governed by galvanic coupling rather than radiative wave propagation.

(2) Geometric configuration: Needle electrodes with an inter-dipole spacing of 2 cm, an implantation depth of 8 mm, and a transmitter-to-receiver separation of 2 cm between the right atrium and right ventricle. Subcutaneous or endocardial placement may alter the current density distribution and fading statistics.

(3) Environmental boundary: An ex vivo isolated porcine heart perfused in a temperature-controlled blood bath. The absence of surrounding thoracic structures (ribs, lungs, pleura), pericardial sac, and dynamic respiratory motion removes potential sources of large-scale shadowing and boundary reflection that could exist in an in vivo setting.

Consequently, the extremely slow-fading classification should not be extrapolated to higher frequencies (>10 MHz), to far-field radio-frequency links, or to in-body geometries involving heterogeneous tissue boundaries without further validation.

### 6.4. Implications for In Vivo Environments

While the ex vivo platform isolates the core cardiac channel physics, several in vivo factors are expected to modify the fading behavior:

(1) Respiratory motion: The diaphragm and chest wall displace the heart by several millimeters to centimeters during the respiratory cycle. This large-scale organ movement introduces periodic variations in the electrode-to-tissue geometry and in the effective transmission distance, potentially generating respiration-induced shadow fading with a coherence time of 3–10 s. Additionally, lung inflation changes the local effective permittivity and conductivity of the surrounding medium, altering the current return path.

(2) Three-dimensional cardiac motion under pericardial constraint: In vivo, the heart undergoes complex twisting, translation, and compression within the pericardial sac. The pericardium and pericardial fluid may shunt a portion of the injected current away from the direct atrioventricular path, introducing an additional parallel impedance branch that is absent in the ex vivo preparation. This could increase the variance of the slow-fading envelope.

(3) Postural changes and blood redistribution: Changes in posture (supine vs. upright) alter the gravitational loading on the heart and the distribution of blood volume within the thorax. This modifies the local hematocrit and tissue conductivity, leading to slow, posture-dependent baseline shifts in channel gain that would manifest as additional shadow fading over time scales of minutes to hours.

These factors suggest that while the intrinsic CIC channel within the myocardial-blood medium is indeed extremely slow-fading, the observed in vivo channel may exhibit modestly larger shadowing variance and, under certain geometric or frequency conditions, weak multipath effects due to the surrounding thoracic anatomy. Future work should incorporate torso phantoms or in vivo telemetry to quantify these extrinsic contributions.

### 6.5. Extension to In Vivo Scenarios

While the ex vivo porcine heart platform provides a controlled and reproducible environment for initial channel characterization, the proposed composite fading model is designed to be extensible to in vivo leadless pacemaker applications. The following aspects highlight how the current model can guide, and be adapted for, in vivo validation.

The frequency-dependent path-loss exponent *n* measured in this ex vivo study captures the intrinsic dielectric properties of cardiac muscle ($\varepsilon_{r}$ and σ). In vivo, additional attenuation arises from the surrounding thoracic tissues (rib cage, lungs, blood perfusion, and adipose layers). Therefore, the absolute path-loss intercept $PL_{0}$ is expected to increase by 5–10 dB, and the effective path-loss exponent *n* may rise moderately.

However, the qualitative trend where channel gain improves with increasing frequency in the 100 kHz–1 MHz band is anticipated to hold, because it is governed by the fundamental dispersion characteristics of muscle tissue rather than the specific geometric configuration. Thus, the ex vivo model provides a lower-bound reference for in vivo link-budget design.

The measured shadow fading in the ex vivo setup is exceptionally weak ($\sigma_{dB}=0.1842$ dB), reflecting the highly stable electrode–tissue interface in a static, blood-free environment. In vivo, this component is expected to exhibit the most dramatic change. Three physiological mechanisms will dominate:

(1) Cardiac mechanical displacement: The beating heart undergoes translation and rotation of several millimeters, causing dynamic variation in the effective electrode spacing and contact pressure.

(2) Respiratory modulation: The diaphragm and chest wall movement alter the electrical path length and surrounding tissue geometry at 0.2–0.4 Hz, introducing a slow-fading component absent in the ex vivo preparation.

(3) Electrode–tissue interface dynamics: Chronic implantation leads to fibrotic encapsulation and acute motion-induced micro-displacements, both of which modulate the contact impedance and thus the received signal strength.

Consequently, we hypothesize that in vivo shadow fading will follow a composite log-normal distribution with a substantially larger standard deviation ($\sigma_{dB}^{(\mathrm{in}\;\mathrm{vivo})}\approx 2$–6 dB). The current ex vivo model therefore serves as a best-case baseline, and in vivo shadowing should be modeled as the superposition of the ex vivo heartbeat-induced component plus additional respiratory and motion-induced terms:

(19) $$\sigma_{dB,\mathrm{total}}^{2}=\sigma_{dB,\mathrm{cardiac}}^{2}+\sigma_{dB,\mathrm{respiratory}}^{2}+\sigma_{dB,\mathrm{micro}\text{-}\mathrm{motion}}^{2}$$

### 6.6. Future Work

The experimental data of this paper are obtained through experiments on ex vivo porcine hearts, which can not fully replicate the physiological environment of an in vivo human heart, while offering high experimental repeatability and cost efficiency.

The channel parameters obtained under controlled conditions in this paper provide a baseline model and measurement method basis for the next in-vivo experiments. Subsequently, by simply expanding this platform into a full-heart blood flow model and implanting a micro receiver-transmitter, preclinical validation can be achieved.

Since this paper focuses on the relative fluctuation of channel gain rather than the absolute delay, and the heart beat cycle is approximately 0.8 s, which is much longer than the time scale for data acquisition and processing, using the timestamp alignment method can meet the requirements for dynamic characteristic analysis. This measurement system does not achieve hardware synchronization at the receiving and transmitting ends, but since the frequency of channel changes is extremely low, this limitation does not affect the analysis of the channel fluctuation range and statistical characteristics.

In clinical practice, cardiac pacemakers are typically implanted within cardiac chambers; however, in most experimental studies on conductive intracardiac communication, transceivers are placed on the epicardial surface, which remains clinically permissible and widely adopted for feasibility and accessibility.

Future work will focus on implanting miniaturized transceivers into live porcine subjects to conduct in vivo experiments, enabling direct comparison and integrated analysis of data obtained from both in vivo and ex vivo settings. Additionally, we aim to capture the full complex signal, including its phase information. Leveraging the periodic modulation of channel gain induced by cardiac motion, we will propose a low-duty-cycle coding or modulation scheme tailored to exploit these dynamic characteristics, thereby facilitating energy-efficient, low-power data transmission for implantable cardiac devices.

## 7. Conclusions

This paper firstly propose a dynamic channel modeling method for CIC based on sinusoidal response and impulse response. We develop a physical simulation and miniaturized measurement setup to measure dynamic cardiac channel. Motion artifacts and real channel fading are systematically separated in an ex vivo experiments. It is able to reveal the true characteristics of the CIC channel under dynamic conditions for the first time. The structure of the composite fading channel model for CIC and its key parameters are presented for the first time.

Currently, several preliminary conclusions regarding the CIC channel have been drawn:

(1) The CIC channel exhibits capacitive characteristics. The fluctuation in channel gain with free electrodes is primarily caused by motion artifacts, whereas fixed electrodes can effectively suppress this interference.

(2) The dynamic channel gain of the CIC varies periodically with the heartbeat, with a signal fluctuation range of less than 1–2 dB. This variation is attributed to the periodic length changes in myocardial tissue.

(3) Under dynamic conditions, the CIC channel presents extremely weak shadow fading, no significant multipath effect, and no measurable Doppler characteristics, classifying it as an extremely slow-fading channel.

## Figures and Tables

**Table 1:** Statistics of Average Gain and Heart Rate for CIC Channels.

Heart rate (beats/min)	40	50	60	70	80	90	100	110	120
Average Gain (dB)	−53.6867	−53.3739	−53.1476	−53.1168	−53.2812	−53.0302	−53.6730	−53.5853	−53.4591
Fluctuation amplitude (dB)	1.3256	1.2013	0.9942	0.9114	1.3913	0.9842	1.2842	1.2013	1.4913

**Table 2:** Statistics of Average Gain and Flow Rate for CIC Channels.

Flow rate (mL/s)	106.67	133.33	160	186.67	213.33	240	260.67
Average Gain (dB)	−53.4392	−53.5852	−53.4526	−53.5533	−53.5536	−53.5171	−53.8756
Fluctuation amplitude (dB)	0.8285	1.2013	0.9114	1.0771	1.4085	1.4913	1.5327

## Data Availability

The data presented in this study are available on request from the corresponding author. The data are not publicly available due to privacy.

## Supplementary Materials

The following supporting information can be downloaded at: https://www.mdpi.com/article/10.3390/bioengineering13060628/s1, Video S1.
